# Smoking Ban in Psychiatric Inpatient Unit: An Iranian Study on the Views and Attitudes of the Mental Health Staff and Psychiatric Patients

**DOI:** 10.1155/2018/2450939

**Published:** 2018-09-17

**Authors:** Narges Beyraghi, Azadeh Mazaheri Meybodi, Reyhaneh Sadat Jafarian Bahri

**Affiliations:** ^1^Department of Psychiatry, University of Toronto, Center for Addiction and Mental Health. Toronto, Canada; ^2^Department of Psychiatry, Shahid Beheshti Medical University, Tehran, Iran; ^3^Master of Clinical Psychology, Taleghani General Hospital, Tehran, Iran

## Abstract

Although the move to smoke-free mental health inpatient settings is an internationally common and popular trend, these policies are neither implemented nor supported by any national program in Iran. This study investigates the attitude of mental health staff and psychiatric patients toward smoking cessation in 2 psychiatric inpatient units (psychosomatic and adult general psychiatry) in the Taleghani general hospital in Tehran. One hundred and twenty participants of this cross-sectional study consist of 30 mental health staff and 90 psychiatric patients. An eight-item questionnaire was used for collecting information. Both staff and patients expressed a positive attitude towards smoking cessation. Patients favoured the implementation of these policies and expressed a more positive attitude towards the feasibility. Sixty-three percent of patients and 57% of staff were opposed to smoking in the units. Seventy percent of patients reported the smoke-free ban as a feasible policy compared to 45% of staff who did the same. The implementation of the smoke-free policy has more support in both staff and patients than the continuation of smoking in psychiatric units. There is a need for an ongoing education and training for mental health care providers, in order to have a successful implementation of smoke-free policy.

## 1. Introduction

It is estimated by some recent studies that the current prevalence of smoking in Iran is 12.5% of the adult population (23.4% males and 1.4% females; burden: 6.1 million) [1]. People with mental illnesses have a higher rate of smoking; this could be explained by several reasons such as stress, self-medication, increased vulnerability to smoking, inaccessibility of health messages, institutional factors, and smoking causative relations with mental disorders [2]. Some studies have shown that psychiatric patients have lower cessation rates compared to the general population [3, 4]. This may be due to a greater relief with smoking and the improvement of some mental health symptoms in comparison with the other population [5, 6].

Although people with mental illness are about twice as likely to smoke as other persons [7], Iranian studies showed that smoking rate was approximately three times higher in psychiatric patients than what has been reported from the general population [8].

Tobacco control policies and cessation interventions that do not target the mentally ill population are not as effective for people with mental disorders [9]. Some studies revealed that the smoking rate in mentally ill smokers who had access to mental health services was not different from those who did not have [10].

In Iran, the tobacco industry is entirely run by the government and no smoking related advertisement is permitted. A number of regulations have been placed in order to decrease tobacco consumption and to ban smoking in public places [11]. Iranian studies reveal that smoking cessation in Iranian health system is a neglected area. The huge gap between ratified laws and performing of laws is mentioned in further advisement. [12, 13]. Despite the availability of effective smoking cessation therapies, as well as the existence of supporting evidence that individuals with psychiatric disorders are motivated to quit, smoking remains a neglected health problem within this patient population [14].

Based on the enacted Iranian National Nicotine Control legislation in 2007, the ministry of Health affirmed administrative regulations that ban smoking in all enclosed public places including hospitals and health settings. But the regulations are still not enforced in mental health settings, and patients continue to smoke inside the units in most of the Iranian psychiatric facilities (behdasht.gov.ir). Patients and mental health staff will benefit from smoke-free policies; however, they may still regard smoking as a normal part of being a psychiatric patient. In order to reach a successful implementation of these policies, we have to overcome many barriers and consider smoke-free policies. This is an opportunity to develop an integrated care model focusing on both the physical and psychological health of mental health patients that will also have benefit for their caregiver [15].

The aim of this study was to assess barriers of change by evaluating the perceptions and attitudes of patients and staff.

## 2. Methods

This cross-sectional study was done in Taleghani General Hospital of Shahid Beheshti Medical University in Tehran, in 2017. Taleghani is a smoke-free hospital except for the two psychiatric units. One of these units is a secure closed/locked general adult psychiatric ward with 24 psychiatric beds, and the other one is an 18-bed open/psychosomatic unit. At the time of this study, patients were allowed to smoke in the closed unit while the open unit had the partial smoking ban rules in place and Nicotine Replacement Therapy (NRT) was offered.

The study sample consisted of 90 psychiatric patients and 30 mental health staff. Psychiatric patients are diagnosed according to the International Classification of Diseases and Related Health Problems (ICD-10). Two separate questionnaires were designed based on a relevant literature review for the patients and staff. Three psychiatrists and two mental health professionals approved the face validity of the questionnaires. Pretesting (two patient- two staff) was done and followed by a minor revision.

Accepting the research consent was the inclusion criteria for both patients and staff and on top of that the patients had to be mentally stable (confirmed by the responsible psychiatry resident). General opinion, the health hazard of smoking, NRT and education, ethical concerns, and feasibility of smoking ban were the main target of the questionnaires. Patients and staff questionnaire are presented in Tables 1 and 2, respectively.

All questionnaires are filled anonymously. Prospectively during a six-month period, new patients and all staff were invited to complete the questionnaire.

To obtain the consent for the study, the clinical psychologist of the unit provided information for patients and staff in one-to-one sessions. Assistance for filling questionnaire was provided based on the participant's request.

All procedures were performed in accordance with the 1964 Helsinki Declaration and its later amendments or comparable ethical standards and informed consent was obtained from all individual participants included in the study. The protocol of study was approved by the ethical committee of the Shahid Beheshti University of medical sciences.

## 3. Results

The sociodemographic status of the participants in both groups is presented in Table 3. Only one of our mental health staff reported being nicotine-dependent (20 cigarettes per day, CPD). Forty-eight patients (53%) reported not to be a current smoker. Cigarette consumption, based on self-report, was 17 patients (18.5%) less than 10, 20 patients (22%) 10-20, and 5 patients (5.5%) more than 20 CPD. It was the first psychiatric admission for 44 of our patients (49%), and 33 (37%) of our participant reported this admission to be their second or third psychiatric admission.

The average staff's work experiences in mental health field was 6 years (Min 1 year–Max 15 years). The staff sample consists of 6 physicians (20%), 4 Masters or Ph.D. (13%), 8 Bachelor (27%), 9 Diploma (30%), and 3 (10%) with high school education.

Results of the questionnaire are presented in Tables 1 and 2. Based on the category of the question and comparison of patients and staff results are as follows.

### 3.1. General Opinion

20% of our staff compared to 23% of patients expressed no concern of being in a smoking unit. 56.7% of our staff and 63% of our patient were against. Others preferred to reply as indifferent.

### 3.2. Physical Health

The staff expressed concern about their own physical health in 89% of cases and in 90% of cases for their patients. Patients also expressed concern about their own and staff's physical health in 78% and 89% of cases, respectively. Only 37% of staff members believed that smoking is psychologically harmful to patients.

### 3.3. Ethical Concern and Smoking Ban Policy

Most of our study population, 87% of staff, and 82% of patients believed that obligatory stop smoking policy is unfair to nicotine dependent patients. 97% of staff and 81% of patients confirmed the need for smoking cessation education and advice [6].

### 3.4. Feasibility of Smoke-Free Policy and Nicotine Replacement Therapy (NRT)

80% of staff and 69% of patients agreed on the feasibility of NRT assisted smoking prevention. Although 63% of patients believed that a smoking ban policy is feasible for psychiatric units, from the staff's point of view only 20% believed in the feasibility of this policy.

## 4. Discussion

To best of our knowledge, the present study is the first study about smoking ban in psychiatric inpatient units in Iran. The study tries to report the views and attitudes of mental health staff and psychiatric patients towards smoking ban in psychiatric inpatient units in a psychiatric service. The results show that the majority of patients and staff are against smoking in the units and agree with the smoke-free policy implementation in psychiatric care settings. This study is an example of psychiatric units in which patients are still smoking indoors despite the enormous evidence of negative health consequences. Current literature shows positive outcomes of implementing smoking cessation policy in psychiatric units [2]. Consistent with previous study results, the finding of this study highlights the mixed sense of resistance and conformity of mental health care provider about smoking in psychiatric units [15]. Future steps towards transitioning to a smoke-free facility may transform the permissive culture and negative rationales of the staff, which previously supported the psychiatric units smoking exemption [16].

The caregivers' permissive attitude expressed in this study could consider a barrier for future implementation of the smoke-free policy [17]. To avoid the future pitfalls, the implementation of smoke-free policies in these acute mental health wards should have a plan for sustained investment in staff training and comprehensive cessation or abstinence support for patients [18]. The type of unit and the population served matters in smoke-free implementation process. Inpatient setting of this study and a very low rate of smoking in the health care providers may contribute to the positive results in perceptions and attitudes [7–19].

Most of staff and patients believed smoking is dangerous for their physical health. Similarly, to other studies in other countries, psychiatric patients agreed that smoking is a dangerous and harmful habit [20–22].

Most of our studied population, 87% of staffs and 82% of patients, believe that an obligatory no-smoking policy is unfair to nicotine-dependent patients. Although the studies of Dickens and Kourakos show that most patients agree with smoking rules and that the staff should encourage them to quit smoking, they state that the staff should avoid smoking in front of the patients [22, 23].

In the present study, most of the staff stated that they should educate and advise patients about quitting smoking. Some studies showed that mental health settings staff could play a powerful role in smoking cessation in psychiatric patients [24]. They can organize a seminar on tobacco cessation and give counselling to help patients with quitting smoking [25, 26]. It is very important that most of the staff were concerned about the dangerous effect of smoking on the mental and physical health the patients, but they believed that an obligatory no-smoking policy is unfair and is a violation of human rights [25, 27, 28]. Mental health staff in these studies believe that all of the patients should make a decision about quitting smoking by themselves and staff responsibility should be limited in smoking cessation, education, and advice.

Other studies show that some sociodemographic factors such as age, income, sex, and education correlated with the patients' attitudes towards smoking cessation [22, 23].

A small percentage of the staff reported a smoke-free ban to be a feasible policy, while most of the patients believed that a smoke-free ban is feasible. The study suggests that patients like to quit smoking but they need help to do so, due to the fact that they cannot quit by themselves. Other studies show that most of the patients agreed that it is too hard for them quit smoking, and a smoking room is not a good choice for them because these rooms will make it difficult for them to quit smoking [8, 22, 23].

Previous researchers have discovered that smoking cessation bans work well amongst patients during their hospital admission, but they expressed various concerns about training mental health staff for the implementation of a smoking ban. The staff's role is very important in delivering smoking cessation services [6, 29–31].

The main limitation of this study was that it took place in only one center with a small sample size. Future researches with samples from many institutions are needed to plan an applicable regional and national policy change. Qualitative studies could complement the survey and would provide more in-depth insights into the barriers to change.

## 5. Conclusions

The present study shows that implementation of a smoke-free policy has more support in both staff and patients in comparison with the continuation of smoking in psychiatric units. Mental health professionals will benefit from education in smoking cessation treatment. There is a need for ongoing education and training of mental health care provider in order to achieve a successful implementation of the smoke-free policy [9].

## Figures and Tables

**Table 1:** View and attitudes about smoking in psychiatric inpatient unit, patients questionnaire (total = 90 patients).

Questions	Agree Frequency/ %	Indifferent Frequency/ %	Disagree Frequency/ %
Q1: I am very upset that patients are smoking in the units	57(63.3%)	21(23.3%)	12(13.3%)

Q2: Smoking in the unit is harmful to my health	70(77.8%)	9(10%)	11(12.2%)

Q3: Smoking in the unit is harmful to staff's health	80(88.9%)	7(7.8%)	3(3.3%)

Q4: It is feasible to quit smoking during psychiatric admission	57(63.3%)	17(18.9%)	16(17.8%)

Q5: It is not fair to force patients to quit smoking during their admission	74(82.2%)	5(5.6%)	11(12.2%)

Q6: We should educate patient and advise patients about quit smoking	73(81.1%)	15(16.7%)	2(2.2%)

Q7: Patients could quit smoking with replacement therapy in the unit	62(68.9%)	21(23.3%)	7(7.8%)

**Table 2:** View and attitudes about smoking in psychiatric inpatient unit, staff's questionnaire (total = 30 staff).

Questions	Agree Frequency/ %	Indifferent Frequency/ %	Disagree Frequency/ %
Q1: I am very upset that patients are smoking in the units	17(56.7%)	6(20%)	7(23.3%)

Q2: I think that working in a smoke allowed unit is harmful to my health	26(86.7%)	2(6.7%)	2(6.7%)

Q3: I think that working in a smoke allowed unit is harmful to patient's health	27(90%)	2(6.7%)	1(3.3%)

Q4: Smoking in the unit is harmful to patient's psychological health	10(33.3%)	7(23.3%)	13(43.3%)

Q5: It is not fair to force patients to quit smoking during their admission	26(86.7%)	1(3.3%)	3(10%)

Q6: We should education patient and advise patients about quit smoking	29(96.7%)	1(3.3%)	0(0%)

Q7: Patients could quit smoking with replacement therapy in the unit	24(80%)	6(20%)	0(0%)

Q8: Changing a psychiatric unit to a smoke-free unit is not feasible	14(46.7%)	10(33.3%)	6(20%)

**Table 3:** Demographic characteristics of health staff and psychiatric patients.

	Mental health staff (N=30)	Psychiatric patients (N=90)
Age mean (SD ±)	40.2 (±8.1)	39.2 (±13.3)

Female/male	11/19	46/44

Education No/%		

*High school or less*	3 (10%)	31 (34.4%)

*High school diploma*	5 (16.7%)	34 (37.8%)

*Bachelor*	12 (40%)	23 (25.6%)

*Masters*	3 (10%)	2 (2.2%)

*MD or PhD*	7 (23.3%)	0 (0.0%)
